# Family-Based Preventive Interventions for Problematic Internet Use Among Children and Adolescents: Protocol for a Systematic Review and Meta-Analysis

**DOI:** 10.3390/ijerph23050637

**Published:** 2026-05-11

**Authors:** Saya Moriyama, Minoru Takahashi, Aimi Hayashi, Takayuki Harada

**Affiliations:** 1 Counseling Science Degree Program, Faculty of Human Sciences, Graduate School of Comprehensive Human Sciences, University of Tsukuba, 3-29-1 Otsuka, Bunkyo-ku, Tokyo 112-0012, Japan; 2 Department of Psychological Counseling, Faculty of Psychology, Mejiro University, 4-31-1 Nakaochiai, Shinjuku-ku, Tokyo 161-8539, Japan; m-takahashi@mejiro.ac.jp; 3 Department of Rehabilitation, IMS Tokyo Katsushika General Hospital, 4-18-1 Nishi-Shinkoiwa, Katsushika-ku, Tokyo 124-0021, Japan; s2340219@u.tsukuba.ac.jp; 4 Institute of Human Sciences, University of Tsukuba, 1-1-1 Tennodai, Ibaraki, Tsukuba 305-8572, Japan; tkharada@human.tsukuba.ac.jp

**Keywords:** problematic internet use, internet addiction, family-based intervention, primary prevention, systematic review, meta-analysis

## Abstract

**Highlights:**

**Public health relevance—How does this work relate to a public health issue?**

- Problematic internet use (PIU) among children and adolescents is increasingly recognized as a public health concern associated with multiple adverse outcomes.
- Family and parenting factors are consistently linked to PIU risk, suggesting a relevant target for preventive interventions.

**Public health significance—Why is this work of significance to public health?**

- Despite growing research on treatment, evidence on preventive interventions—particularly those involving families—remains limited and fragmented.
- This protocol aims to systematically identify and evaluate family-based preventive interventions for PIU in youth.

**Public health implications—What are the key implications or messages for practitioners, policy makers and/or researchers in public health?**

- This review will provide evidence on whether family-based approaches are associated with improved prevention outcomes.
- Findings may inform future research and the development of prevention strategies to support healthy internet use in young populations.

**Abstract:**

Problematic internet use among children and adolescents is a major public health concern. In this protocol, it is defined as construct encompassing problematic gaming, social media use, smartphone use, and undifferentiated use, characterized by impaired control, prioritization, and persistence despite harm. It is associated with academic, sleep, and psychosocial difficulties. However, preventive interventions—particularly family-based—remain underexplored despite evidence linking parenting and family functioning to risk. This protocol outlines systematic review and meta-analysis of family-based preventive interventions among children and adolescents (6–18 years). Randomized controlled, quasi-randomized, cluster-randomized, and quasi-experimental studies with parallel comparison groups will be included. Comparators defined as no intervention, waitlist, usual care, or non-family-based prevention. Searches will be conducted in CENTRAL, PubMed, PsycINFO, Web of Science, and CiNii Research, supplemented by reference screening. Risk-of-bias will be assessed using RoB 2 and ROBINS-I. Primary outcomes include changes in overall and subtype-specific severity; secondary outcomes include use time, family functioning, and parental involvement. Random-effects meta-analyses with Hartung–Knapp adjustment will be conducted when ≥3 homogeneous studies are available; otherwise, findings will be synthesized narratively following the Synthesis Without Meta-analysis guideline. This review will synthesize current evidence and clarify role of family-based prevention, informing research and public health strategies.

## 1. Introduction

Internet use is integral to the daily lives of children and adolescents, and concerns about problematic internet use (PIU) have increased. PIU is broadly understood as a pattern of internet use characterized by impaired control over online activities and continued use despite negative consequences [1]. It involves diverse online activities, including gaming, social media use, smartphone-mediated internet use, streaming, online shopping, and other internet-based behaviors. Because PIU encompasses heterogeneous behaviors, measurement traditions, and levels of clinical recognition, it remains difficult to define and measure uniformly [2]. Accordingly, this protocol treats PIU as an umbrella construct while explicitly classifying related behavioral phenotypes. Recent syntheses suggest that PIU and related behaviors are prevalent and clinically relevant among youth [3,4]. A global meta-analysis estimated the pooled prevalence of internet addiction at approximately 7% in the general population, rising to roughly 9% among Japanese junior high school students [5,6]. Notably, the prevalence of Internet gaming disorder and PIU has been suggested to be higher in Asian countries, including Japan, than in Europe and North America [7]. Additionally, PIU has been associated with poor academic performance [8], sleep problems [9], and other psychosocial difficulties among youth [10,11]. Among PIU-related behaviors, gaming disorder has received formal nosological recognition in the International Classification of Diseases, 11th Revision (ICD-11), where it is defined by impaired control, prioritization of gaming over other activities, and continued use despite negative consequences [12]. Recent evidence further indicates that PIU-related subtypes remain clinically and public health relevant among young people. For example, a systematic review and meta-analysis reported associations between problematic social media use and symptoms of depression, anxiety, and stress among adolescents and young adults [13] and another estimated the burden of gaming disorder among adolescents [14].

Within this umbrella construct, PIU-related outcomes will be classified into overlapping but distinguishable behavioral phenotypes, including problematic (disordered) gaming, problematic social media use, problematic smartphone use, and general or undifferentiated PIU. These subtypes share core features of impaired control, prioritization of the online activity over other domains of functioning, and continued use despite negative consequences [1,4]. They nevertheless differ in primary digital medium, measurement tradition, and nosological status. Gaming Disorder is formally recognized in the ICD-11 [12], whereas problematic social media use and problematic smartphone use are generally treated as research constructs operationalized through validated psychometric scales [13,15,16]. To preserve this conceptual hierarchy, eligible studies will be classified by phenotype at data extraction, and phenotype will be examined as a prespecified subgroup and, where data permit, through subtype-specific meta-analyses.

From a prevention perspective, family and parenting processes are strongly associated with PIU risk among youth, including internet-specific practices (e.g., rule setting, monitoring), general parenting style, and family functioning [17,18,19]. Recent evidence further supports the role of family context. A systematic review reported associations between parenting-related factors and adolescents’ problematic social media use [20], and a prospective study showed that internet-specific parental rules may relate to its onset [21]. In Japan, attachment styles have also been associated with PIU among adolescents [22]. These correlational findings, together with prevention theory, suggest that interventions targeting parents and families—rather than the child alone—may reduce PIU risk before problems become entrenched.

Although numerous treatment programs for PIU have been developed and evaluated, particularly among adolescents, preventive approaches have received comparatively less attention [23,24]. Recent reviews indicate that preventive interventions for internet addiction and problematic digital technology use are emerging, particularly in school- and child-focused settings; however, they remain heterogeneous in target population, delivery format, intervention components, and outcome measures [25,26,27].

Clinical and intervention research further highlights the importance of family-based approaches in addressing PIU. A randomized controlled trial (RCT) targeting adolescents who met the criteria for Internet gaming disorder (IGD) in the Diagnostic and Statistical Manual of Mental Disorders, Fifth Edition (DSM-5) demonstrated that multidimensional family therapy reduced IGD prevalence and achieved high retention rates [28]. More recent evidence, including a meta-analysis of family-based therapy for internet addiction among adolescents and young adults, suggests that family-oriented approaches may reduce symptom severity [29]. However, this evidence primarily concerns treatment rather than prevention. Although family-based approaches are increasingly recognized as important, existing evidence remains insufficient to determine the effectiveness of such preventive interventions.

This review is informed by two complementary theoretical perspectives. A family systems perspective [30,31] conceptualizes the family as an interdependent unit in which parent–child relationships, family cohesion, and communication patterns may shape individual behavior, suggesting that sustained change in a child’s digital behavior requires change at the family level. Bronfenbrenner’s bioecological model [32] further positions the family microsystem as the proximal developmental context in which children acquire self-regulation and media habits, nested within broader school, community, and cultural systems. Taken together, these frameworks provide a rationale for prioritizing interventions that actively engage parents and caregivers rather than targeting the child alone, and they guide our a priori categorization of intervention components (e.g., parent–child communication, shared rule-setting, co-use, parental monitoring).

Against this backdrop, there is a clear need for an up-to-date synthesis that quantifies the effects of family-based preventive interventions for PIU and related behaviors among school-age children and adolescents, while examining how delivery format and prevention level influence outcomes. Although the number of rigorous preventive trials may still be limited, this reflects the emerging nature of the field rather than the absence of public health need. PIU-related problems have become increasingly salient alongside rapid changes in children’s digital environments, and preventive strategies remain insufficiently established. By systematically organizing the available evidence at this stage, this review aims to provide a foundation for future research, intervention development, and public health strategies. This protocol addresses this need by specifying transparent methods for identifying and synthesizing evidence on family involvement in preventing youth PIU.

### Objectives

The primary objective is to estimate the efficacy of family-based preventive interventions for PIU among children and adolescents (aged 6–18 years) compared with (a) no intervention, waitlist, or usual care and (b) preventive programs without family participation.

The secondary objectives are to investigate differences in the effectiveness of family-based preventive interventions by delivery format (face-to-face vs. online), level of risk, sex, and age subgroup.

## 2. Methods

This protocol follows the Preferred Reporting Items for Systematic Reviews and Meta-Analyses Protocols (PRISMA-P) statement and checklist [33]. A completed PRISMA-P checklist is provided as an additional file and referenced in the manuscript. The protocol was registered with the International Prospective Register of Systematic Reviews (PROSPERO) and will be updated as required [34] (registration ID: CRD420251124901, last updated: 16 October 2025). This study will be conducted between October 2025 and March 2027.

### 2.1. Criteria for Considering Studies for This Review

#### 2.1.1. Types of Studies

RCTs, quasi-RCTs, cluster-RCTs, and quasi-experimental studies with clearly defined parallel comparison groups will be included.

#### 2.1.2. Types of Participants

Children and adolescents aged 6–18 years recruited from any setting (e.g., schools, communities, clinics) will be eligible. No restrictions will be applied regarding sex, socioeconomic status, or geographical location. Regarding baseline PIU status, we will include studies enrolling (i) general-population samples without pre-selection for PIU risk (universal prevention), (ii) at-risk samples defined by exposure to known risk factors or subclinical elevation on a validated PIU screener (selected prevention), and (iii) samples showing early signs of PIU—operationalized as scores on a validated PIU screener at or above a published at-risk cut-off but below a clinical-range cut-off, or documented subclinical concerns reported by the participant or caregiver, without a formal clinical diagnosis (indicated prevention). Studies enrolling participants who already meet DSM-5 or ICD-11 criteria for Gaming Disorder or clinically diagnosed Internet Use Disorder, or those that exclusively deliver treatment to diagnosed samples, will be excluded.

#### 2.1.3. Types of Interventions

For this review, a family-based preventive intervention is operationalized as any structured program in which at least one parent, caregiver, or other family member participates as an active agent of change—not merely as a passive informant or recipient of information about the child—delivered through one or more sessions targeting family-level mechanisms relevant to PIU, including parent–child communication, co-regulation of digital media use, shared rule-setting, monitoring or mediation, parent skills training, family education, or joint parent–child activities. Programs in which parents are only notified of outcomes, receive leaflets, or provide consent without active participation will not be classified as family-based. Interventions may be delivered in any format (group, individual, face-to-face, or online), at any level of intensity or duration.

Following the Institute of Medicine classification [35], each included intervention will be categorized as universal, selected, or indicated prevention. Studies evaluating clinical treatment for diagnosed Gaming Disorder or Internet Use Disorder will be excluded; studies combining indicated prevention and treatment components will be included only if data for the preventive (subclinical) arm can be extracted separately.

#### 2.1.4. Types of Comparators

Comparators will include no intervention, waitlist control, usual care, or preventive interventions without family involvement.

#### 2.1.5. Types of Outcomes

##### Primary Outcomes

The primary outcome is the change in PIU symptoms from baseline, measured using a validated psychometric instrument reported in the included study. To preserve the subtype hierarchy described in the Introduction, PIU-related outcomes will be classified into four behavioral phenotypes: (a) overall or undifferentiated PIU, measured with instruments such as the Internet Addiction Test [36] or the Compulsive Internet Use Scale [37]; (b) problematic gaming, measured with instruments such as the Game Addiction Scale for adolescents [38]; (c) problematic social media use, measured with instruments such as the Bergen Social Media Addiction Scale [39]; and (d) problematic smartphone use, measured with instruments such as the Smartphone Addiction Scale [40]. Where a study reports more than one phenotype, each will be extracted. Subtype-specific meta-analyses will be conducted when at least three studies are available per phenotype; otherwise, the phenotype will be modeled as a prespecified subgroup moderator within a combined analysis, and subtype-specific findings will be presented narratively.

##### Secondary Outcomes

Secondary outcomes include:

- Children’s internet use time, measured as self-reported or parent-reported average daily or weekly internet use (hours).
- Family functioning (e.g., cohesion, communication), measured using validated instruments such as the Family Adaptability and Cohesion Evaluation Scales [41] or the Family Assessment Device [42].
- Parental involvement and monitoring, assessed using validated scales such as the Parental Monitoring Scale or other established parental mediation measures.

#### 2.1.6. Information Sources and Search Strategy

Electronic databases to be searched include the Cochrane Central Register of Controlled Trials (CENTRAL), PsycINFO, PubMed, Web of Science, and CiNii Research. The reference lists of the included studies will also be screened manually to identify additional eligible studies. Complete database-specific search strategies are provided in Supplementary Material S2. To enhance reproducibility, the core conceptual blocks and a representative PubMed search strategy are presented below. The search strategy combines four conceptual blocks: (1) children and adolescents, (2) PIU and related behavioral subtypes, (3) family or parental involvement, and (4) intervention or prevention, using the Boolean structure: (population) AND (PIU) AND (family) AND (intervention/prevention). The representative PubMed strategy is as follows: (Adolescent[Mesh] OR Child[Mesh] OR adolescent*[tiab] OR teen*[tiab] OR youth[tiab] OR child*[tiab] OR student*[tiab]) AND (“internet addiction”[tiab] OR “problematic internet use”[tiab] OR “internet use disorder”[tiab] OR “gaming disorder”[tiab] OR “internet gaming”[tiab] OR “problematic gaming”[tiab] OR “problematic social media”[tiab] OR “social networking addiction”[tiab] OR “smartphone addiction”[tiab] OR “problematic smartphone”[tiab] OR “mobile phone dependence”[tiab]) AND (parent*[tiab] OR caregiver*[tiab] OR famil*[tiab] OR mother*[tiab] OR father*[tiab] OR “parent–child”[tiab] OR parenting[tiab] OR “parental mediation”[tiab] OR “parental monitoring”[tiab]) AND (intervention*[tiab] OR prevent*[tiab] OR program*[tiab] OR training[tiab] OR education[tiab] OR “randomized controlled trial”[pt] OR “controlled clinical trial”[pt] OR trial*[tiab]). Equivalent strategies adapted to the syntax and controlled vocabulary of each database will be executed in CENTRAL, PsycINFO, Web of Science, and CiNii Research; Japanese-language terms will be added for CiNii Research.

Records will be limited to reports published in English or Japanese. This restriction reflects two considerations. First, the review team’s working languages are English and Japanese, enabling accurate eligibility assessment, data extraction, and risk-of-bias evaluation. Second, existing RCTs of PIU prevention and family-based interventions have predominantly been published in English, and our scoping searches identified no eligible RCT outside these literatures that was not also indexed in English. The number of records excluded on language grounds will be transparently reported, and the language restriction will be discussed as a potential source of bias in the final review.

Scoping searches conducted between September and October 2025 returned approximately 1200 unique records across the five databases after preliminary deduplication. Approximately 15–25 studies are expected to meet the eligibility criteria after title/abstract and full-text screening. This estimate will be updated in the final review.

### 2.2. Study Records

#### 2.2.1. Data Management

Citations will be managed and deduplicated using a reference manager. Title/abstract and full-text screening will be conducted using a web-based screening tool that records decisions. Data extraction will be conducted using standardized forms (e.g., spreadsheets or databases), piloted on a sample of studies to ensure consistency.

#### 2.2.2. Selection Process

Two review authors will independently screen the abstracts of all retrieved studies, resolving any disagreements through discussion. Subsequently, full-text copies of all potentially relevant studies will be retrieved, and assessed independently for eligibility by two review authors. Any disagreements will be resolved through discussion and, if necessary, by a third review author.

#### 2.2.3. Data Collection Process

At least two review authors will independently extract the data using a predesigned data extraction form, including study design, participant characteristics, intervention details, control conditions, and outcomes. Any discrepancies will be resolved through discussion or, if required, consultation with a third author. If any information is unclear, trial authors will be contacted for further details.

#### 2.2.4. Data Items

- Study characteristics: country, setting, design, sample size, recruitment methods, inclusion and exclusion criteria.
- Participant characteristics: age, sex, baseline PIU status/risk.
- Intervention details: family involvement (who/how), components (e.g., parent training, communication skills development), delivery mode (face-to-face or online), dose (sessions, duration), facilitator background.
- Comparator details: content or intensity.
- Outcomes: instrument names, scoring, time points; means or standard deviations (for continuous variables) or counts (for dichotomous variables); analysis set (intention-to-treat/completers).

### 2.3. Assessment of Risk of Bias in Included Studies

Two reviewers will independently assess risk of bias using Risk of Bias 2 (RoB 2) for randomized trials and Risk of Bias in Non-randomized Studies of Interventions (ROBINS-I) for non-randomized studies, with consensus and third-reviewer adjudication as needed. Domain-level judgments and overall risk of bias per outcome will be reported.

### 2.4. Data Analysis

A PRISMA flow chart of study search and selection will be reported, and excluded studies will be presented with reasons for exclusion [43]. Extracted data will be presented in tables, alongside a narrative synthesis summarizing study design, participant characteristics, interventions, outcome measures, and risk of bias assessments for each outcome, enabling comparison across studies.

#### 2.4.1. Conditional Analytic Pathway

Analytic steps are conditional on the volume and homogeneity of the retrieved evidence. When at least three studies report sufficiently homogeneous outcomes (same behavioral phenotype, comparable comparator, conceptually related measurement), effects will be pooled quantitatively. Otherwise, findings will be synthesized narratively following the Synthesis Without Meta-analysis (SWiM) reporting guideline [44], using structured tabulation, effect-direction plots, and, where appropriate, vote-counting based on direction of effect.

#### 2.4.2. Effect Estimation and Pooling

A meta-analysis of the primary outcome will be performed using R version 4.5.1 (R Foundation for Statistical Computing, Vienna, Austria) with the meta and metafor packages [45], with RevMan Web (The Cochrane Collaboration, London, UK) [46] used for cross-checking. Continuous outcomes will be summarized using standardized mean differences (Hedges’ *g*) with 95% confidence intervals, and pooled using a random-effects model with Hartung–Knapp adjustment to account for between-study heterogeneity. For dichotomous outcomes, risk ratios (preferred) or odds ratios will be calculated. Separate meta-analyses will be conducted for post-intervention and longest follow-up outcomes. Forest plots will be generated for all pooled analyses.

#### 2.4.3. Subgroup, Moderator, and Small-Study Analyses

Thresholds for additional analyses are prespecified to avoid overfitting in a potentially sparse evidence base. If sufficient data are available, subgroup analyses will be performed when at least five studies are available per comparison, by behavioral phenotype (problematic gaming, social media, smartphone, or undifferentiated PIU), delivery format (face-to-face vs. online), prevention level (universal, selected, or indicated), age group (children vs. adolescents), sex, and risk of bias (low vs. some/high). Meta-regression moderator analyses—including intervention duration, follow-up length, and baseline risk—will be performed when at least ten studies are available per outcome. When ≥10 studies are included, funnel plots will be visually inspected, and Egger’s test and trim-and-fill methods will assess small-study effects. Sensitivity analyses will be performed by excluding high-risk-of-bias studies, variation of intraclass correlation coefficient (ICC) assumptions for cluster trials, and conducting leave-one-out influence analyses. For cluster-randomized trials, effective sample sizes will be calculated using reported or externally sourced ICCs. Multi-arm trials will be combined or split to avoid double counting, in line with Cochrane guidance. All statistical codes and outputs will be archived to support reproducibility.

#### 2.4.4. Strategies for Managing Heterogeneity

Clinical, methodological, and statistical heterogeneity are anticipated and will be addressed through a prespecified three-tiered strategy. Clinical heterogeneity will be addressed by stratifying meta-analyses by phenotype (problematic gaming, social media use, smartphone use, or undifferentiated PIU) and by prevention level (universal, selected, indicated). Methodological heterogeneity will be addressed by analyzing randomized and non-randomized designs separately, with pooled RCT-only estimates reported as primary results and non-randomized evidence presented as sensitivity analyses. Statistical heterogeneity will be quantified using I^2^ and τ^2^, and interpreted alongside prediction intervals. When I^2^ exceeds 75% or prediction intervals span both clinically meaningful benefit and harm, pooled estimates will be reported with explicit caution, and the synthesis will emphasize narrative interpretation in line with SWiM [44].

### 2.5. Meta-Bias

If ≥10 studies are available for a given outcome, publication bias will be visually assessed using funnel plots and statistically tested using Egger’s regression test. If asymmetry is detected, the trim-and-fill method will be applied to estimate its impact. Selective outcome reporting will be evaluated by comparing published reports with trial registrations or protocols, where available, and any discrepancies will be documented. Information on funding sources and declared conflicts of interest will also be extracted and considered for each included study.

#### 2.5.1. Confidence in Cumulative Evidence

We will assess the certainty of evidence for key outcomes using the Grading of Recommendations Assessment, Development, and Evaluation approach and present a Summary of Findings table. Any decisions to downgrade or upgrade the certainty of evidence will be reported transparently and justified.

#### 2.5.2. Amendments

Important protocol amendments (e.g., changes to eligibility criteria, outcomes, or analytical methods) will be documented with the date, rationale, and a clear description of the change. All amendments will be reported in the final review and updated in the PROSPERO registration record.

## 3. Discussion

This protocol outlines a systematic review and meta-analysis designed to synthesize the existing evidence on family-based preventive interventions for PIU among children and adolescents. The primary objective is to determine whether programs involving parents or caregivers are effective in reducing PIU-related symptoms and risks compared with no intervention or preventive programs without family participation.

The significance of this review lies in its focus on family-based prevention, an approach that remains relatively underexplored yet is theoretically important. Previous research has demonstrated associations between parenting practices, family functioning, and youth PIU [17,18,19,20,21,22], suggesting that family-level interventions may be more effective than those targeting individuals alone [28,29]. By systematically synthesizing trials that incorporate family factors, this review will help clarify how such interventions may support healthy patterns of internet use during critical developmental periods.

A major strength of this protocol is its comprehensive design, which includes both randomized trials and quasi-experimental studies, as well as the use of standardized tools to assess risk-of-bias. By examining differences in delivery format, prevention level, and demographic subgroups, the review may yield insights into potential effect modifiers and inform adaptation across contexts.

Several limitations should be acknowledged. First, the concept of PIU is heterogeneous, and variation in outcome measures may complicate data synthesis. Second, the limited number of rigorous trials may restrict the generalizability of the findings. Finally, while the inclusion of quasi-experimental designs broadens the evidence base, it may also increase the risk of bias, necessitating cautious interpretation of the results.

In summary, this review aims to provide timely and methodologically rigorous evidence on the role of families in preventing PIU among children and adolescents. The findings are expected to inform future interventions and contribute to policies and practices that promote healthy digital engagement in youth.

## Data Availability

No new data were generated in this study protocol. Data extracted from included studies and statistical analysis code will be made available upon reasonable request to the corresponding author.

## Supplementary Materials

The following supporting information can be downloaded at: https://www.mdpi.com/article/10.3390/ijerph23050637/s1, Supplementary File S1: PRISMA-P 2015 checklist [33,47]; Supplementary File S2: Search Strategy.

## Abbreviations

The following abbreviations are used in this manuscript:

CENTRAL: Cochrane Central Register of Controlled Trials; ICC: Intraclass correlation coefficient; IGD: Internet gaming disorder; PIU: Problematic internet use; PRISMA-P: Preferred Reporting Items for Systematic Review and Meta-Analysis Protocols; RCT: Randomized controlled trial; ROBINS-I: Risk of Bias in Non-randomized Studies of Interventions; RoB 2: Risk of Bias 2; SWiM: Synthesis Without Meta-analysis.
