# Supplementary material for: Family-Based Preventive Interventions for Problematic Internet Use Among Children and Adolescents: Protocol for a Systematic Review and Meta-Analysis
Source: Int J Environ Res Public Health. 2026 May 11;23(5):637. doi: 10.3390/ijerph23050637 (PMC13206802; doi:10.3390/ijerph23050637)
Supplement: Supplementary file 1 [file ijerph-23-00637-s001.zip › ijerph-4269907-supplementary/Supplementary Files/S2_Search Strategy.pdf]

Supplementary Material S2 — Search Strategy

1. PubMed

Search interface. PubMed advanced builder with field tags [Mesh], [tiab], and [pt]. MeSH terms are exploded by default.

Search execution date. To be recorded at execution.

#1 Population

Adolescent[Mesh] OR Child[Mesh] OR adolescent*[tiab] OR teen*[tiab]
OR youth[tiab] OR child*[tiab] OR student*[tiab] OR "young people"[tiab]
OR "young persons"[tiab] OR pediatric*[tiab] OR paediatric*[tiab]
OR schoolchild*[tiab]

#2 Problematic Internet Use and subtypes

"Internet Addiction Disorder"[Mesh] OR " "internet addiction"[tiab]
OR "problematic internet use"[tiab] OR "internet addiction"[tiab]
OR "internet use disorder"[tiab] OR "pathological internet use"[tiab]
OR "compulsive internet use"[tiab] OR "excessive internet use"[tiab]
OR "gaming disorder"[tiab] OR "internet gaming disorder"[tiab]
OR "internet gaming"[tiab] OR "problematic gaming"[tiab]
OR "excessive gaming"[tiab] OR "video game addiction"[tiab]
OR "social media addiction"[tiab]
OR "problematic social media use"[tiab]
OR "social networking addiction"[tiab]
OR "problematic social networking"[tiab]
OR "smartphone addiction"[tiab] OR "problematic smartphone use"[tiab]
OR "problematic smartphone"[tiab] OR "excessive smartphone use"[tiab]
OR "mobile phone addiction"[tiab] OR "mobile phone dependence"[tiab]
OR "cell phone addiction"[tiab]

#3 Family or parent involvement

Parents[Mesh] OR Family[Mesh] OR "Parent-Child Relations"[Mesh]
OR Caregivers[Mesh] OR parent*[tiab]
OR famil*[tiab] OR caregiver*[tiab] OR mother*[tiab] OR father*[tiab]
OR "parent child"[tiab] OR "parent-child"[tiab] OR parenting[tiab]
OR "parental mediation"[tiab] OR "parental monitoring"[tiab]
OR "family-based"[tiab] OR "family based"[tiab]

#4 Intervention or prevention

"Primary Prevention"[Mesh] OR "Health Promotion"[Mesh]
OR prevent*[tiab] OR "health promotion"[tiab] OR intervention*[tiab]
OR program*[tiab] OR programme*[tiab] OR training[tiab]
OR education[tiab] OR "randomized controlled trial"[pt]
OR "controlled clinical trial"[pt] OR trial*[tiab]

#5 #1 AND #2 AND #3 AND #4

Filters applied to #5:

Language: English OR Japanese

Publication type: not restricted (all study designs eligible at the search stage; non-empirical records will be excluded at title/abstract screening)

Date: not restricted at the search stage; date stratification will be performed in sensitivity analyses.

2. Cochrane Central Register of Controlled Trials (CENTRAL)

Search interface. Cochrane Library, advanced search, MeSH and free-text fields combined.

Search execution date. To be recorded at execution.

#1 MeSH descriptor: [Adolescent] explode all trees

#2 MeSH descriptor: [Child] explode all trees

#3 (adolescent* OR teen* OR youth OR child* OR student* OR
"young people" OR "young persons" OR pediatric* OR paediatric*
OR schoolchild*):ti,ab,kw

#4 MeSH descriptor: [Internet Addiction Disorder] explode all trees

#5 ("problematic internet use" OR "internet addiction" OR
"internet use disorder" OR "pathological internet use" OR
"compulsive internet use" OR "excessive internet use" OR
"gaming disorder" OR "internet gaming disorder" OR
"internet gaming" OR "problematic gaming" OR
"excessive gaming" OR "video game addiction" OR
"social media addiction" OR "problematic social media use" OR
"social networking addiction" OR "problematic social networking" OR

"smartphone addiction" OR "problematic smartphone use" OR
"problematic smartphone" OR "excessive smartphone use" OR
"mobile phone addiction" OR "mobile phone dependence" OR
"cell phone addiction"):ti,ab,kw

#6 MeSH descriptor: [Parents] explode all trees
#7 MeSH descriptor: [Family] explode all trees
#8 MeSH descriptor: [Parent-Child Relations] explode all trees
#9 MeSH descriptor: [Caregivers] explode all trees
#10 (parent* OR famil* OR caregiver* OR mother* OR father* OR
"parent child" OR "parent-child" OR parenting OR
"parental mediation" OR "parental monitoring" OR
"family-based" OR "family based"):ti,ab,kw
#11 MeSH descriptor: [Primary Prevention] explode all trees
#12 MeSH descriptor: [Health Promotion] explode all trees
#13 (prevent* OR "health promotion" OR intervention* OR
program* OR programme* OR training OR education OR
trial*):ti,ab,kw

#14 (#1 OR #2 OR #3) AND (#4 OR #5) AND (#6 OR #7 OR #8 OR #9
OR #10) AND (#11 OR #12 OR #13)

Limits: Trials only (CENTRAL is by definition a controlled-trials register,
so no further design filter is needed); Language English or Japanese.

3. PsycINFO (APA PsycNet)
Search interface. APA PsycNet advanced search, with APA Thesaurus terms and free-text fields. Thesaurus terms are exploded.
Search execution date. To be recorded at execution.
S1 DE "Adolescent Development" OR DE "Childhood Development" OR
DE "Adolescent Psychology" OR DE "Child Psychology"
S2 TI(adolescent* OR teen* OR youth OR child* OR student* OR
"young people" OR pediatric* OR paediatric* OR schoolchild*)
OR AB(adolescent* OR teen* OR youth OR child* OR student* OR
"young people" OR pediatric* OR paediatric* OR schoolchild*)
S3 S1 OR S2

S4 DE "Internet Addiction" OR DE "Computer Game Addiction" OR
DE "Mobile Phone Use" OR DE "Online Social Networks" OR

S5 TI("problematic internet use" OR "internet addiction" OR
"internet use disorder" OR "pathological internet use" OR
"compulsive internet use" OR "excessive internet use" OR
"gaming disorder" OR "internet gaming disorder" OR
"problematic gaming" OR "excessive gaming" OR
"video game addiction" OR "social media addiction" OR
"problematic social media use" OR "social networking addiction" OR
"smartphone addiction" OR "problematic smartphone use" OR
"excessive smartphone use" OR "mobile phone addiction" OR
"mobile phone dependence" OR "cell phone addiction")
OR AB(same terms as TI)

S6 S4 OR S5

S7 DE "Parents" OR DE "Family" OR DE "Parent-Child Relations" OR
DE "Caregivers" OR DE "Parental Involvement"

S8 TI(parent* OR famil* OR caregiver* OR mother* OR father* OR
"parent-child" OR parenting OR "parental mediation" OR
"parental monitoring" OR "family-based")
OR AB(same terms as TI)

S9 S7 OR S8

S10 DE "Prevention" OR DE "Health Promotion" OR DE "Intervention" OR
DE "Treatment Effectiveness Evaluation"

S11 TI(prevent* OR "health promotion" OR intervention* OR
program* OR programme* OR training OR education OR trial*)
OR AB(prevent* OR "health promotion" OR intervention* OR
program* OR programme* OR training OR education OR trial*)

S12 S10 OR S11

S13 S3 AND S6 AND S9 AND S12

Limits: Language: English OR Japanese.

4. Web of Science Core Collection

Search interface. Web of Science basic search; Topic field (TS=) covers title, abstract, author keywords, and Keywords Plus. Editions: SCI-EXPANDED, SSCI, A&HCI, ESCI.

Search execution date. To be recorded at execution.

#1 TS=(adolescent* OR teen* OR youth OR child* OR student* OR
"young people" OR "young persons" OR pediatric* OR paediatric*
OR schoolchild*)

#2 TS=("problematic internet use" OR "internet addiction" OR
"internet use disorder" OR "pathological internet use" OR
"compulsive internet use" OR "excessive internet use" OR
"gaming disorder" OR "internet gaming disorder" OR
"internet gaming" OR "problematic gaming" OR
"excessive gaming" OR "video game addiction" OR
"social media addiction" OR "problematic social media use" OR
"social networking addiction" OR "problematic social networking" OR
"smartphone addiction" OR "problematic smartphone use" OR
"excessive smartphone use" OR "mobile phone addiction" OR
"mobile phone dependence" OR "cell phone addiction")

#3 TS=(parent* OR famil* OR caregiver* OR mother* OR father* OR
"parent child" OR "parent-child" OR parenting OR
"parental mediation" OR "parental monitoring" OR
"family-based" OR "family based")

#4 TS=(prevent* OR "health promotion" OR intervention* OR
program* OR programme* OR training OR education OR trial*)

#5 #1 AND #2 AND #3 AND #4

Refine by:

Languages = ENGLISH OR JAPANESE

Document Types = (no restriction; non-empirical records will be
excluded at title/abstract screening)

5. CiNii Research

Search interface. CiNii Research advanced search; Japanese-language keywords combined with free-text terms.

Search execution date. To be recorded at execution.

The CiNii Research interface supports a single search box; the strategy below is constructed by entering each block as a separate query and intersecting result sets.

ブロック 1 — 対象集団（Population）:
児童 OR 子ども OR 子供 OR 青少年 OR 思春期 OR 青年期 OR
小学生 OR 中学生 OR 高校生 OR 学生 OR 生徒
（English-language equivalents also entered: child*, adolescent*,
youth, student*）

ブロック 2 — 問題使用（PIU and subtypes）:
ネット依存 OR インターネット依存 OR ネット中毒 OR
ゲーム障害 OR ゲーム依存 OR
スマートフォン依存 OR スマホ依存 OR 携帯依存 OR
SNS 依存 OR ソーシャルメディア依存
（English-language equivalents also entered: "internet addiction",
"problematic internet use", "gaming disorder",
"smartphone addiction", "social media addiction"）

ブロック 3 — 家族・保護者（Family/parent involvement）:
家族 OR 家庭 OR 保護者 OR 親 OR 母親 OR 父親 OR
養育者 OR 家族療法 OR 家族支援 OR 親子関係 OR ペアレンティング
（English-language equivalents also entered: family, parent*,
caregiver*, "parent-child", parenting）

ブロック 4 — 介入・予防（Intervention/prevention）:
予防 OR 介入 OR プログラム OR 教育 OR 訓練 OR
ランダム化比較試験 OR RCT
（English-language equivalents also entered: prevention,
intervention*, program*, training, education, trial*）

ブロック 1 AND ブロック 2 AND ブロック 3 AND ブロック 4
